# Comparative Analysis of DNA Methyltransferase Gene Family in Fungi: A Focus on Basidiomycota

**DOI:** 10.3389/fpls.2016.01556

**Published:** 2016-10-21

**Authors:** Ruirui Huang, Qiangqiang Ding, Yanan Xiang, Tingting Gu, Yi Li

**Affiliations:** ^1^State Key Laboratory of Plant Genetics and Germplasm Enhancement and College of Horticulture, Nanjing Agricultural UniversityNanjing, China; ^2^Laboratory of Plant Hormone, College of Life Sciences, Nanjing Agricultural UniversityNanjing, China; ^3^Department of Plant Science and Landscape Architecture, University of ConnecticutStorrs, CT, USA

**Keywords:** DNA methyltransferase, basidiomycetes, *Pleurotus ostreatus*, mushroom development, DNA methyltransferase inhibitor

## Abstract

DNA methylation plays a crucial role in the regulation of gene expression in eukaryotes. Mushrooms belonging to the phylum Basidiomycota are highly valued for both nutritional and pharmaceutical uses. A growing number of studies have demonstrated the significance of DNA methylation in the development of plants and animals. However, our understanding of DNA methylation in mushrooms is limited. In this study, we identified and conducted comprehensive analyses on DNA methyltransferases (DNMtases) in representative species from Basidiomycota and Ascomycota, and obtained new insights into their classification and characterization in fungi. Our results revealed that DNMtases in basidiomycetes can be divided into two classes, the Dnmt1 class and the newly defined Rad8 class. We also demonstrated that the fusion event between the characteristic domains of the DNMtases family and Snf2 family in the Rad8 class is fungi-specific, possibly indicating a functional novelty of Rad8 DNMtases in fungi. Additionally, expression profiles of DNMtases in the edible mushroom *Pleurotus ostreatus* revealed diverse expression patterns in various organs and developmental stages. For example, DNMtase genes displayed higher expression levels in dikaryons than in monokaryons. Consistent with the expression profiles, we found that dikaryons are more susceptible to the DNA methyltransferase inhibitor 5-azacytidine. Taken together, our findings pinpoint an important role of DNA methylation during the growth of mushrooms and provide a foundation for understanding of DNMtases in basidiomycetes.

## Introduction

Basidiomycota (basidiomycetes) account for around 32% of fungi and are important to forestry, agriculture, and medicine (Riley et al., 2014). This phylum includes mushrooms, pathogens of plants, and animals, and other fungi (Riley et al., 2014). Mushrooms are highly important for both nutritional and pharmaceutical uses (Wani et al., 2010). One characteristic feature of mushrooms is that they can form a fruiting body and produce basidiospores during sexual reproduction. However, research into the role played by epigenetic modification in mushroom development is limited. *Pleurotus ostreatus* has been widely studied for its edible fruit body, short life cycle, and easy cultivation (Bonatti et al., 2004). In addition, genome sequences of *P. ostreatus* are available (Riley et al., 2014). Hence, it is a readily accessible model organism for research exploring the role of DNA methylation in mushroom development.

DNA methylation refers to the transfer of an activated methyl group (CH_3_) from S-adenosyl methionine to the 5th position of a cytosine residue (5mC, Law and Jacobsen, 2010). This reaction is catalyzed by DNA methyltransferases (DNMtases) with the DNA methylase domain as the catalytic domain. In both animals and fungi, 5mC happens on the CG context (Ehrlich et al., 1982). Two distinct classes of DNA methyltransferases, DNMT1 and DNMT3, have been reported in mammals. DNMT1 is responsible for the maintenance of DNA methylation, ensuring proper inheritance of the methylation pattern following DNA replication in somatic cells. By contrast, DNMT3s (3A, 3B) are required for *de novo* methylation of previously unmethylated sequences, contributing to the establishment of DNA methylation patterns during embryogenesis. In plants, 5mC happens on CHG and CHH (H = A, C, or T) context as well (Henderson and Jacobsen, 2007). Three types of DNMtases have been reported in plants, including DNA METHYLTRANSFERASE 1 (MET1) essential for the maintenance of 5mC on the CG context, the plant-specific CHROMOMETHYLASE 3 (CMT3) responsible for the maintenance of 5mC in the CHG context, and DOMAINS REARRANGED METHYLTRANSFERASE 1 and 2 (DRM1/2) responsible for *de novo* methylation at all sequence context (Law and Jacobsen, 2010). DNMT2 is another class of DNA methylase domain-containing enzymes, which are responsible for tRNA^Asp^ methylation in all kingdoms (Jeltsch et al., 2006; Zemach and Zilberman, 2010; Zemach et al., 2010).

DNA methylation is necessary for normal development in both mammals and plants. In mice, knockout mutation of either *Dnmt1* or *Dnmt3b* exhibit lethality during embryonic development (Li et al., 1992; Okano et al., 1999). In *Arabidopsis thaliana*, reduction in DNA methylation leads to floral abnormalities (Finnegan et al., 2000). Additionally, in *Neurospora crassa*, DNA methylation is crucial for the balancing between the formation of sexual and asexual reproductive structures (Filippovich et al., 2004).

Bisulfite sequencing showed that methylation at CG sites is a major pattern in fungi, and that DNA methylation often occurs in transposable elements (TEs) and undergoes global reprogramming during fungal development (Zemach et al., 2010; Jeon et al., 2015). Two distinct genome defense processes for TEs are found in ascomycetes including Repeat-Induced Point mutation (RIP) and Methylation Induced Premeiotically (MIP). These two mechanisms require the specific *de novo* DNA methyltransferases RID and Masc1 of the Dnmt1 superfamily, respectively (Goyon and Faugeron, 1989; Malagnac et al., 1997). Furthermore, *de novo* methylation has been reported in *C. cinereus* (a model basidiomycete species). *De novo* methylation in *C. cinereus* shares many features with MIP in *A. immerses* (Kües, 2000). However, key proteins required for MIP in *C. cinereus* remain enigmatic (Lee et al., 2008).

Although extensive studies concerning DNA methylation have been performed in plants, animals, and ascomycete fungi (mostly molds and yeasts), little attention has been paid to studying how DNA methylation is involved in basidiomycete development, particularly in mushrooms (Martienssen and Colot, 2001; Goll and Bestor, 2005). Therefore, identification and characterization of DNA methylation modifiers would be of significance for understanding epigenetic regulatory mechanisms underlying development of basidiomycetes. An increasing number of basidiomycete genomes have been sequenced (Riley et al., 2014), offering an unprecedented opportunity to unravel the classification and potential functions of DNA methyltransferases in basidiomycetes. Here we sampled 38 representative fungal species, from Mucoromycotina and Dikayons (which includes Basidiomycota and Ascomycota) and conducted an extensive analysis with regard to the classification and characterization of their DNA methyltransferases. We identified four DNMtases in *P. ostreatus* and performed a comprehensive study on structures and expression patterns exhibited during the growth of mushroom. Additionally, 5-azacytidine treatment of monokaryons and dikaryons further confirmed that DNA methylation plays a more important part in the growth of dikaryons than in that of monokaryons. This study paves the way for elucidation of structure and function of DNMtases in basidiomycetes.

## Materials and methods

### Data sampling

For DNA methylase domain containing proteins, we selected a total of 39 different whole-genome sequenced species, including 38 fungi and one mammalian as comparison (for more details see Supplementary Tables 1, 2). Their complete genomic sequences (for sequence IDs see Supplementary Table 1) and annotation information were downloaded from online databases National Center for Biotechnology Information (NCBI; http://www.ncbi.nlm.nih.gov/assembly/) and Joint Genome Institute (JGI; http://genome.jgi.doe.gov/).

For each species, protein entries matching the DNA methylase domain (PF00145) in the Pfam database v28.0 (Finn et al., 2013) were identified as DNA methyltransferase-encoding genes using HMMER searches with an *e*-value cut-off of 10^−4^ (Eddy, 1998). To verify the DNA methylase domain, the outputs were also analyzed by the Conserved Domain Database (CDD; http://www.ncbi.nlm.nih.gov/Structure/cdd/wrpsb.cgi available from NCBI) searches and the Simple Modular Architecture Research Tool database (SMART; http://smart.embl-heidelberg.de/), with a threshold of *e* < e^−4^ (Letunic et al., 2015; Marchler-Bauer et al., 2015).

Likewise, apart from five fungi species, 18 different whole-genome sequenced species, including four eubacteria, three archaebacteria, three protists, four plants, and four animals were sampled for domain fusion event investigation (detailed in Supplementary Table 2). The complete protein sequences of these 18 species were downloaded from NCBI. Protein entries matching the SNF2_N domain (PF00176) and Helicase_C domain (PF002716) in the Pfam database v28.0 (Finn et al., 2013) were identified for further analysis using HMMER searches with an *e*-value cut-off of 10^−4^ (Eddy, 1998).

### Multiple sequence alignment and tree building

DNA methylase domains identified were aligned with MUSCLE v3.8.31 with default parameters (Edgar, 2004) and the alignment was subsequently adjusted manually. Before tree building, the model was selected by ModelGenerator v0.85 (Keane et al., 2006). ML phylogenetic trees were constructed using MEGA 6.0, using the WAG model of evolution from 1000 replicates to evaluate the reliability of internal branches. The other parameters were kept as default settings.

### Analysis of deduced proteins

Protein sequences were analyzed for domain organization using Pfam (http://pfam.xfam.org/search) and EvolView (http://www.evolgenius.info/evolview/#login). In addition, motif analysis was performed online by MEME (MEME, Version4.10.2, http://meme-suite.org/tools/meme) (Bailey and Elkan, 1994). The maximum number of motifs was set at 20 with the length from 10 to 50 amino acids for each search. Protein structures were predicted by the Phyre2 program (Kelley and Sternberg, 2009) and viewed by Chimera1.10.2. Specific protein interactions were constructed applying STRING software (Search Tool for the Retrieval of Interacting Genes/Proteins, http://string-db.org/) (Szklarczyk et al., 2011).

### Material collection and preparation

The *P. ostreatus* material was provided by Jiangsu Academy of Agricultural Science. Mycelium of *P. ostreatus* was collected by scraping material with a sterile spatula from PDA culture medium incubated at 25°C for 10 days. Organs and tissues from different developmental stages were harvested from a first-flush crop according to Hammond (Hammond and Nichols, 1975). The samples were immediately frozen in liquid nitrogen and stored at −80°C before use.

### Total RNA isolation and cDNA synthesis

Total RNA was extracted from fungi materials using a plant RNA mini Kit (Qigen, German) according to the manufacturer's protocol. Both A260/A230 and A260/A280 ratios for all samples were measured to ensure the purity. The quality was confirmed by the electrophoresis 1% (wt/vol) agarose gels. RNase-free DNase was used to remove potential genomic DNA contamination according to the manufacturer's instructions (TaKaRa, Dalian, China). A total of 1 μg of RNA of each sample was used to synthesize first strand cDNA using M-MLV reverse transcriptase according to the manufacturer's instructions (TaKaRa, Dalian, China). The cDNA samples were diluted 10-fold for quantitative real-time PCR (qRT-PCR) analysis.

### Quantitative PCR analyses

To analyze DNMtases gene transcript levels in different developmental stages and in hypha after the 5-azacytidine treatment, we performed qRT-PCR on an Applied Biosystems 7500 real-time PCR system using a SYBR Green RT-PCR kit (Novland, Shanghai, China). The primers used for relative quantification were designed from PrimerQuest Tool of IDT database with CDS sequence of PC15 version 2.0. All the primers used in this experiment are provided in Supplementary Table 4. All primers used for relative quantification were synthesized by Genescript Nanjing Inc. (Nanjing, China). Results were analyzed by using the ΔΔCT (Livak and Schmittgen, 2001) method using ACTIN (Castanera et al., 2012) as the internal control. Three biological replicates and three technical replicates were performed and analyzed. All data were processed with SPASS 20.0 for Windows using Ducan's multiple range teat at the *P* < 0.05 level of significance.

### Hypha growth and chemical treatments

The conidia were collected from the mature sporocarp of *P. ostreatus* and incubated on Petri dishes for 14 days. Monokaryotic and dikaryotic mycelia were identified microscopically according to the clamp connection of edible fungi (Kües, 2000). The mother culture was incubated for 10 days and a small amount of mother culture with fungal mycelia whose diameter is 0.5 cm was inoculated to the new medium with variable concentrations of 5-azacytidine (Sigma). 5-azacytidine in aqueous solution was added to the PDA medium before solidifying at final concentrations of 100, 250, and 500 μM of 5-azacytidine. In all experiments the cultivation was at 25°C in dark and three biological replicates were used. The dikaryotic hypha treated was incubated for 10 days, while the monocaryon hypha was incubated for 14 days due to the slower growth rate. Mycelia growth was visualized by OLMPUS BX43 microscope system and pictures were taken by Canon EOS 700D.

## Results

### Identification of genes containing the DNA methylase domain in fungi

To better understand the classification and evolution of DNMtases in basidiomycetes, we selected 30 representative basidiomycete species, 7 representative ascomycete species and 1 species in Mucoromycotina. To identify DNMtase genes, the full alignment of the DNA methylase domain (PF00145) downloaded from Pfam (http://pfam.xfam.org/search) was searched against proteomes sampled with the HMMER toolset. Using this method, we identified 119 proteins from 38 fungal species and compared them with four canonical DNMtases found in humans (Hermann et al., 2004; Suetake et al., 2004). We named each newly identified protein based on the published names of the closest homolog in humans and followed by a species abbreviation. If two or more fungal homologs from the same species were equally close to a single human gene, then the same name was used followed by the letters “a,” “b,” etc. For the Rad8 family specifically, we named the proteins based on the published names in *C. cinereus*.

### Classification and distribution of putative methyl transferases in fungi

**Figure 1:**
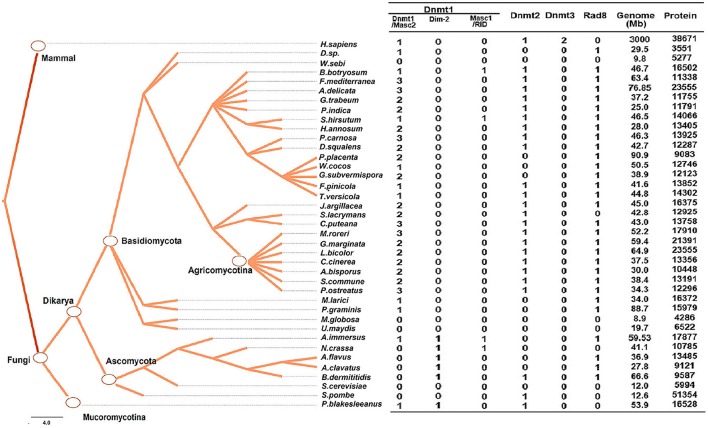
**Numbers of genes encoding DNA methylase domain-containing proteins in the 39 species investigated**. The Taxonomy Common Tree constructed by Taxonomy Browser in NCBI (National Center for Biotechnology Information) is shown on the left. The number detail of each category of each species is listed at the right column. The species lacking gene encoding DNA methyltransferase were also verified by online tblastn toolset of NCBI.

**Figure 2:**
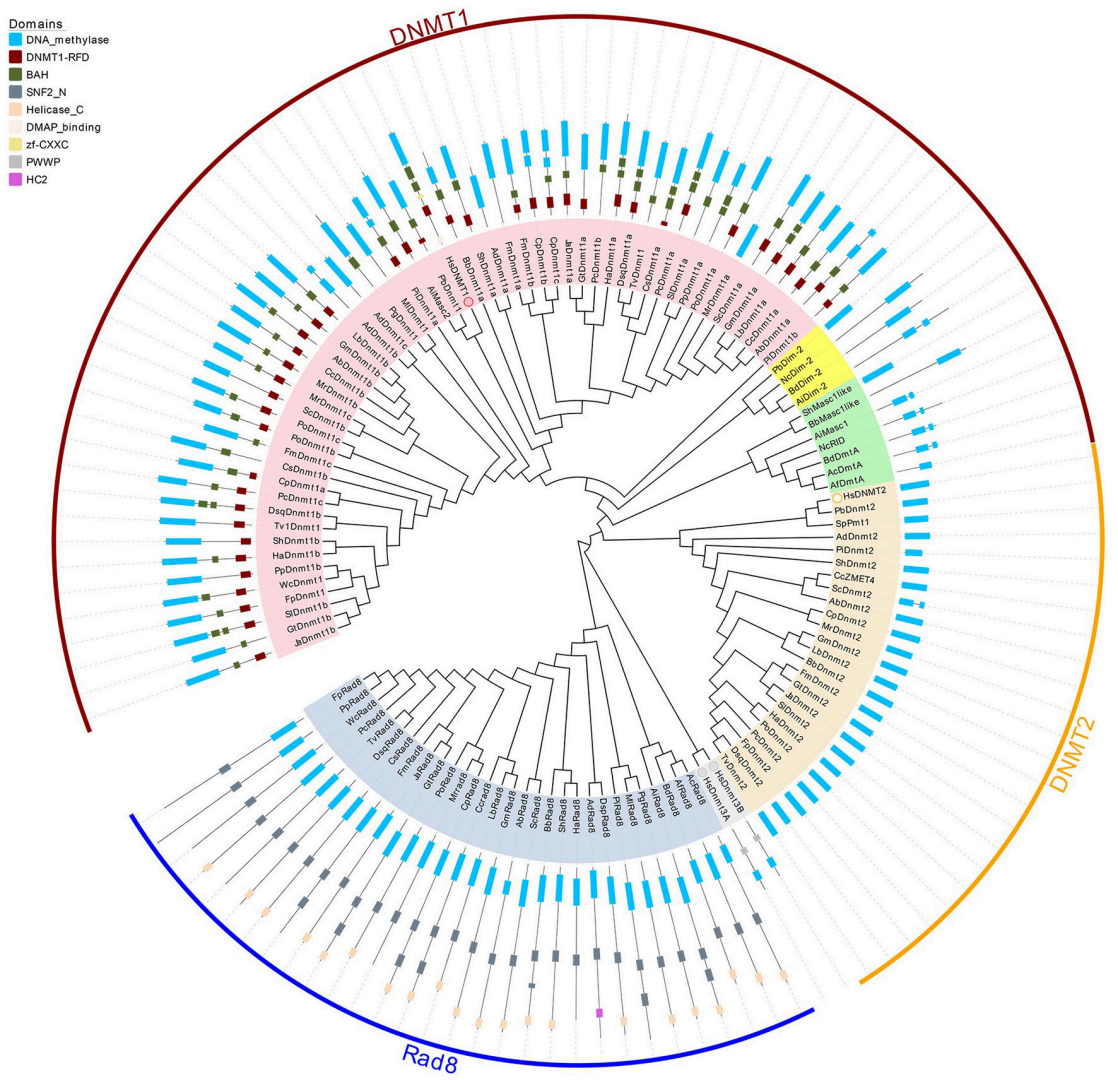
**Phylogenetic and domain architecture analysis of DNA methylase domain containing proteins in fungi**. The ML tree was constructed with the conserved catalytic domains of the predicted 123 proteins from 39 species (listed in Supplementary Table 1) using the MUSCLE v3.8.31 and MEGA6.0 with 1000 bootstrapping replicates. The three families (Dnmt1, Dnmt2, and Rad8) and 3 subfamilies (Dnmt1/Masc2, Dim-2, Masc1/RID) are marked in different colors. The domain architecture analysis of the identified proteins was performed by the Pfam database.

To investigate the classification of putative DNA methyltransferase genes, phylogenetic trees were constructed using the maximum likelihood method (see Section Methods for more details) based on the conserved DNA methylase domain of 123 methyltransferases identified in 39 species (**Figure 2**). Based on these phylogenetic trees, DNA methylase domain-containing proteins in selected fungal species can be roughly grouped into the Dnmt1, Rad8, and Dnmt2 subfamilies (Figure 1, Supplementary Figure 1). The Dnmt1 proteins in fungi were further spilt into three subgroups (Figure 1, Supplementary Figure 1).

The distribution of methyltransferases exhibited several common characteristics among the fungi investigated. First, all fungi analyzed possessed at least one DNMtase (Dnmt1 or Rad8 family), except *Ustilago maydis, Saccharomyces cerevisae, Malassezia globosa*, and *Wallemia sebi* (Figure 1). We verified the absence of DNA methyltransferases in these species using the TBLASTN toolset against the genomic sequences. These species belong to different taxonomic clades, indicating that the lack of DNMtase in some fungi stems from independent losses over evolutionary history. Second, in basidiomycetes, each genome has only one gene copy of the Dnmt2 and Rad8 subfamilies, while containing many copies of the Dnmt1 subfamily (Figure 1). Third, the presence of Dnmt1 and Dnmt2 homologs in both Dikarya (Ascomycota and Basidiomycota) and Mucoromycotina reflects that these two classes of methyltransferases were present in the last common ancestor of all fungi. By contrast, Rad8 proteins exist in Dikarya (both Basidiomyceta and Ascomycota) but not Mucoromycotina (Figure 1).

### Dnmt1 subfamily

The Dnmt1 subfamily can be further divided into three distinct subgroups, Dnmt1/Masc2, Dim-2, and RID/Masc1, based on phylogenetic analysis (Figure 2). In the kingdom Fungi, Dnmt1/Masc2 class DNMTases are present in basidiomycetes, ascomycetes, and *P. blakesleeanus*, which diverged from the Dikarya (ascomycetes and basidiomycetes) more than 1 billion years ago (Hedges et al., 2006, Figure 1). This suggests that Dnmt1/Masc2s are well conserved among fungi.

Domains are considered the basic unit of proteins, thus domain construct analysis was performed by Pfam and further verified by comparisons with the CDD and SMART databases. As illustrated in Figure 2, in addition to the catalytic domain, almost all Dnmt1/Masc2 proteins have similar domain architectures, bearing the DNMT1-RFD and BAH domains, indicating functional similarity among Dnmt1/Masc2s. Furthermore, we conducted motif analyses of the DNA methylase domain and found that similar motif compositions were embedded in the catalytic domain. Specifically, motif 2, 3, 5, 9, 15, 17 are highly conserved in Dnmt1/Masc2s indicating that these motifs might be essential for the function of Dnmt1/Masc2s (Supplementary Figure 1). Genes involved in the same biological process often exhibit coordinated expression, and information regarding their co-expression thus allows further understanding of the biological process in question (Eisen et al., 1998). Subsequently, functional network analysis was carried out to further investigate the candidate proteins that interact with Dnmt1/Masc2s. Protein interaction network analysis demonstrated that both the Dnmt1a in *L. bicolor* and the Dnmt1a in *C. cinereus* might act together with histone deacetylases (Supplementary Figure 2). This indicates that the Masc2s in fungi might also recruit histone deacetylases as DNMT1 does in mammals (Fuks et al., 2000). Taken together, these results show that the Masc2/Dnmt1 subgroup proteins are highly similar among fungi and mammals both in protein structure and possible biological function.

Dim-2 proteins are present in Ascomycota and Mucoromycotina but are absent in Basidiomycota (Figure 1), suggesting that Dim-2 was lost after the radiation of the Dikarya. It is noteworthy that unlike the Dnmt1/Masc2 subgroup, Dim-2 catalyzes DNA methylation in all sequence contexts (Kouzminova and Selker, 2001). Domain architecture analysis and motif composition analysis both identified protein sequence differences between the Dnmt1/Masc2 and Dim-2 subfamilies (Figure 2, Supplementary Figure 1), suggesting a possible functional difference between these two subfamilies. The Masc1 and RID required for MIP and RIP, respectively, has been identified in ascomycetes (Amselem et al., 2015). However, basidiomycetes have neither of these two gene homologs in their genomes (Figures 1, 2), indicating that the genes required for the RIP/MIP pathway might appear after the bifurcation between ascomycetes and basidiomycetes.

### Rad8 subfamily

Rad8 proteins failed to cluster together with any human DNMtase homologs, in contrast to the Dnmt1 and Dnmt2 subfamilies. The DNA methylase domain is essential for the catalytic activity of DNMtase proteins, therefore the Multiple EM for Motif Elicitation (MEME) motif website search program was used to identify the conserved motifs embedded in DNA methylase domains of the Rad8 subfamily. Motif composition analysis of DNA methylase domains showed that motif composition differed between the Dnmt1 and Rad8 subfamilies (Supplementary Figure 1). For instance, motifs 3, 5, and 9 which are highly conserved in the Dnmt1 subfamily, were not identified in the Rad8 subfamily (Supplementary Figure 1). This indicates that those motifs might not be essential for the catalytic activity of the Rad8 proteins (Figure 2). Therefore, it is possible that functional differences between Dnmt1 and Rad8 subfamilies exist.

We further performed domain structure analysis to gain functional insights into the Rad8 subfamily (Figure 2). In addition to the conserved DNA methylase domain, the majority of Rad8 proteins have the SNF2_N domain and the Helicase_C domain (Figure 2). SNF2_N and Helicase_C are two characteristic domains of the Snf2 superfamily, which consists of ATP-dependent chromatin remodeling proteins (Flaus, 2006). Distinct from the Dnmt1 subfamily in both the motif composition of the catalytic domain and the domain architectures, the Rad8 subfamily is a DNA methyltransferase group not previously reported. The domain reorganization event between the DNA methyltransferase and Snf2 families might indicate functional novelty of Rad8 DNMTases.

### The fusion event of characteristic domains between DNMTase and Snf2 families is specific to fungi

**Figure 3:**
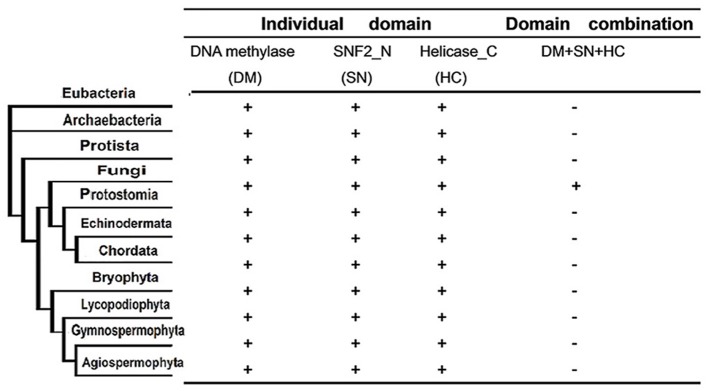
**Phyletic distribution of taxonomic organisms and domain fusion event of characteristic domains between DNMtase family and Snf2 family identified in a genome-wide survey of representative species**. The distribution and domain fusion event were mapped to the phyletic tree. Plus (+) and minus (−) indicate the domain presence/absence in the corresponding phylogenetic clade under the cut-off of *e* < 10^−4^ in the hmmpfam toolset.

Next, we investigated whether the domain fusion event between the characteristic domains of the DNMTase (DNA methylase domain) and Snf2 families (SNF2_N domain and Helicase_C domain) occurred in other kingdoms or solely in fungi. To address this question, we identified all of the proteins constituting the individual domains of Rad8 proteins based on a Pfam homologous search in 23 representative species from six taxonomic kingdoms (Supplementary Table 3). Interestingly, all of the characteristic domains can be detected in Eubacteria and Archaebacteria (Figure 3, Supplementary Table 3), suggesting ancient origins of these functional modules of the DNA methyltransferase and Snf2 families. However, genes encoding proteins with the DNA methylase, the SNF2_N and the Helicase_C domains are only present in the fungal branch (Figure 3). A scan of all current sequence data for the organisms sampled using BLAST from NCBI further confirmed this observation (data not shown). Therefore, the domain fusion event of the DNMtase family and Snf2 family is fungi specific, indicating possible functional novelty of the Rad8 subfamily in fungi.

### Expression profiles of DNMtase genes in *P. ostreatus*

**Figure 4:**
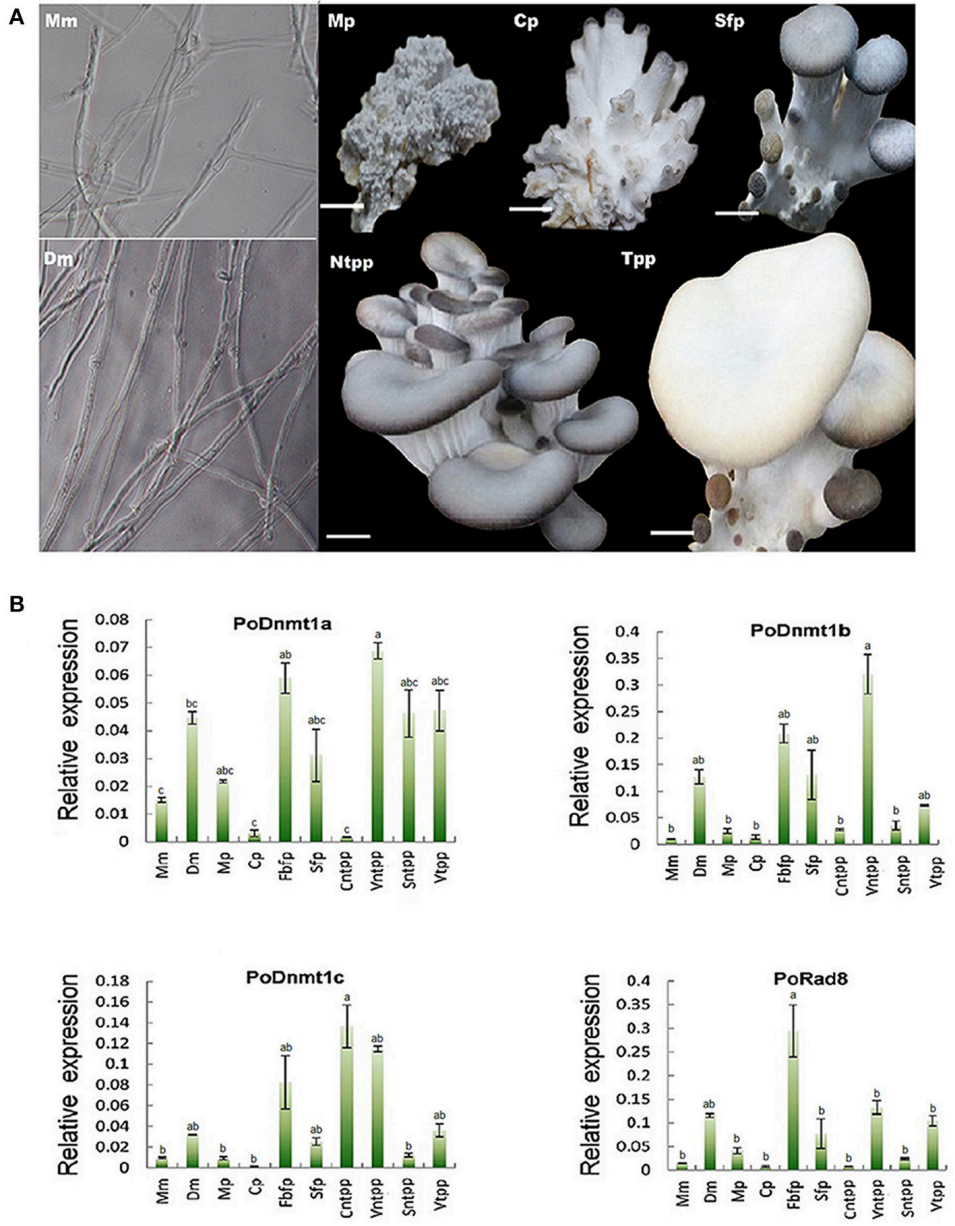
**Expression profiles of DNMtase genes in ***Pleurotus ostreatus***. (A)** The schematic diagram for different developmental stages of *Pleurotus ostreatus*. Mm, Dm, Mp, Cp, Sfp, Ntpp, and Tpp represents monokaryotic mycelium, dikaryotic mycelium, mulberries phase, coral-like phase, sporophor formative phase, not turning pale phase, and turning pale phase, respectively. **(B)** Expression profiles of DNMtase genes during the development of *Pleurotus ostreatus*. The expression levels relative to ACTIN were measured by quantitative RT-PCR and displayed in log2 scale. The acronyms from left to the right are representative of Monokaryotic mycelium (Mm), Dicaryotic mycelium (Dm), Mulberries phase (Mp), Coral-like phase (Cp), Fruiting body of formative phase (Fbfp), Stip of formative phase (Sfp), Cap of not turning plae phase (Cntpp), Veil of not turning pale phase (Vntpp), Stip of not turning pale phase (Sntpp), Veil of turning pale phase (Vtpp) that were collected for quantitative RT-PCR analysis. Three biological replicates and three technological replicates were done for each data point. Error bars indicate standard deviations (SDs). Letters indicate significant differences at *P* < 0.05 according to Duncan's multiple range tests.

DNA methylation is important for the development of mammals, plants, and filamentous fungi (Martienssen and Colot, 2001). To investigate how DNMtases are involved in the development of basidiomycetes, we performed qRT-PCR to investigate the mechanisms of DNMtases regulation during growth of *P. ostreatus*. Here, we sampled different organs or tissues from six key developmental stages (Figure 4A). As shown in Figure 4B, DNMtases generally displayed higher expression levels during reproductive growth than during vegetative growth, indicating that these genes might play a role in the growth of reproductive organs, such as the fruiting body. All of the putative DNMtase genes exhibited low expression in the primordial stage, a decisive differentiation point for the transition from vegetative growth to reproductive growth, suggesting that DNMtase genes may not play a role in the transition process (Figure 4B). As for Dnmt1 homologs, Dnmt1a and Dnmt1b had similar expression patterns, reaching their peaks at the veil of not turning pale phase (Figure 4B). However, Dnmt1c reached its peak at cap maturation (Figure 4B). Rad8 appears to be exclusively and highly expressed in the stage of fruiting body forming (Figure 4B). Intriguingly, the expression levels of DNMtases in dikaryons were significantly higher than those in monokaryons (Figure 4B). In summary, expression profiles of those DNMtase genes in *P. ostreatus* suggest that DNA methyltransferases play organ-specific and stage-specific roles during the growth of the mushroom.

### Dikaryons are more sensitive to 5-azacytidine than monokaryons

**Figure 5:**
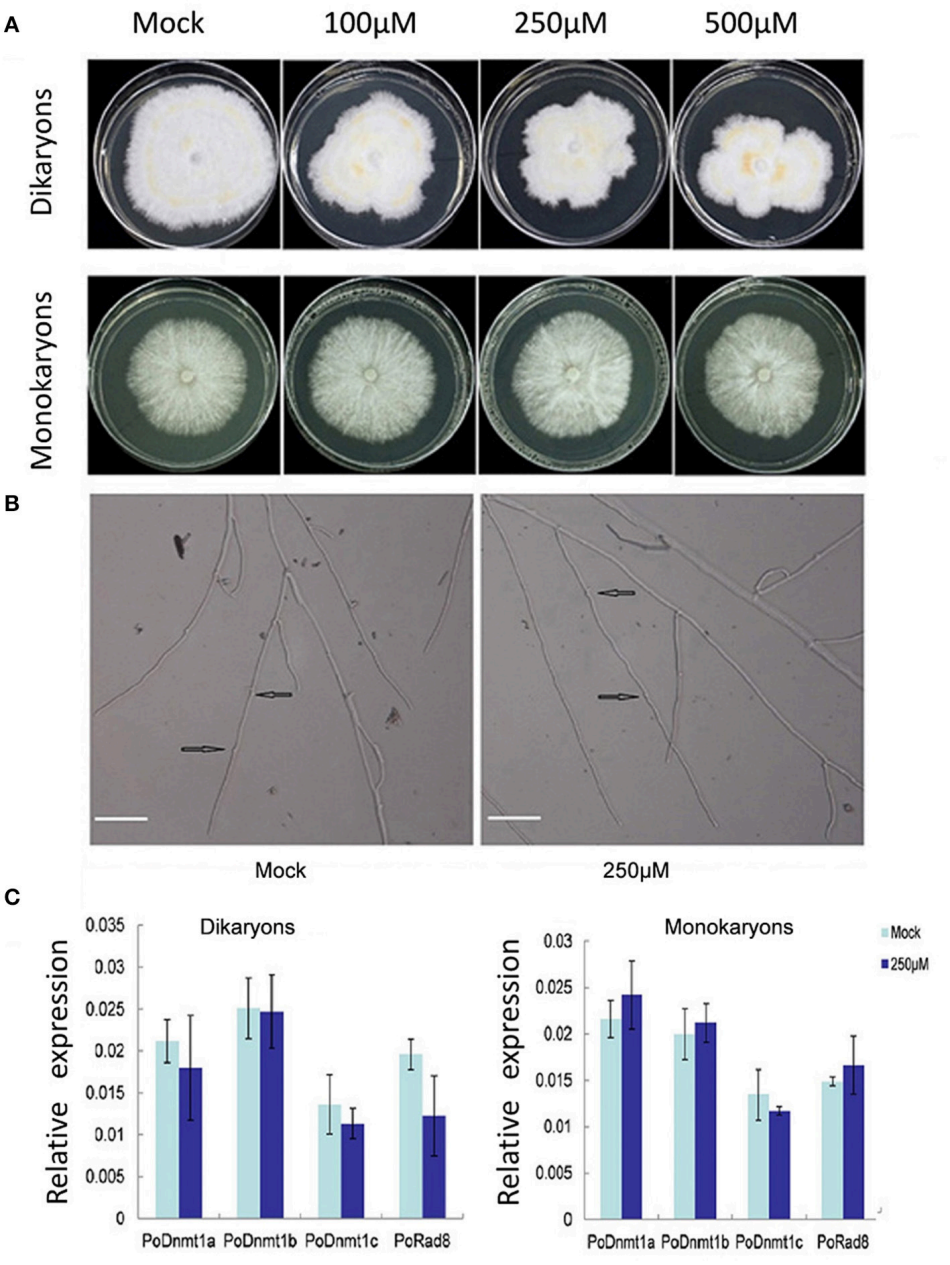
**Dikaryons are more sensitive to the inhibitory effect of 5-azcytidine than monokaryons**. **(A)** Dikaryons and monokaryons grown on 5-azacytidine -containing medium with 100, 250, or 500 μM 5-azacytidine. Images of dikaryons and monokaryons were taken 10, 14 days after inoculation, respectively. **(B)** The cell length of 5-azacytidine treated dikaryons is longer than that of the mock. The arrow indicates where the clamp connection forms. **(C)** Expression profiles of putative DNMtase genes in dikaryons and monokaryons after 5-azacytidine treatment for 10, 14 days. The values shown are means ±SD.

Expression profiles showed that all putative DNA methytranferase genes were expressed at higher level in dikaryons than in monokaryons (Figure 4B). To investigate whether DNMtases have any effects on the growth of dikaryons and monokaryons, we used 5-azacytidine (an inhibitor of DNA methyltransferases) to treat both monokaryons and dikaryons. Monokaryons and dikaryons were incubated on media containing 100, 250, or 500 μM 5-azacytidine. The monokaryons incubated with any concentration of 5-azacytidine (100, 250, or 500 μM) grow in a similar manner to the mock group, with similarly extended colony morphology and branching terminal hypha (Figure 5A, Supplementary Figure 3). However, dikaryons showed inhibited growth, at 100, 250, or 500 μM 5-azacytidine, with less radial extension and a smaller mycelial colony area than the control did (Figure 5A). Of note, at 250 and 500 μM 5-azacytidine, the inhibitory effect on dikaryons was more significant than that at 100 μM, demonstrating that growth inhibition is dose-dependent (Figure 5A). Additionally, levels of secondary metabolites (yellow) of 5-azacytidine treated dikaryons are higher than those of the control (Figure 5A), indicating that 5-azacytidine might affect the mycelial metabolism. Upon microscopic examination, we found that terminal dikaryons treated with 5-azacytidine showed a longer distance between adjacent clamp-connections where new septa will appear (Figure 5B), suggesting that 5-azacytidine can affect cell length. Collectively, these data demonstrate that dikaryons are more susceptible to the inhibitory effect of 5-azacytidine than are monokaryons, which suggests that DNA methylation may play a more essential role in the growth of dikaryons than that of monokaryons.

To investigate whether the different behaviors of monokaryons and dikaryons were resulted from any difference in DNMtase gene expression under 5-azacytidine treatment, the transcript levels of DNMtase genes were investigated. In general, none of the DNMtases showed any expression difference at 250 μM 5-azacytidine relative to control in either monokaryons or dikaryons (Figure 5C). The insensitivity of DNMtase gene expressions upon 5-azacytidine treatment in both monokaryons and dikaryons indicates that DNMtase genes themselves are not regulated by DNA methylation, and that the inhibitory effect of 5-azacytidine on the growth of dikaryons might be resulted from expression changes of other genes important for dikaryon cells.

## Discussion

In this study, we performed a systematic phylogenetic analysis of DNA methylase domain-containing proteins in fungi, with a focus on basidiomycetes. Then we examined their expression patterns in the representative mushroom species, *P. ostreatus*. We identified a total of 119 DNA methylase domain-containing proteins from 38 fungi, and divided them into Dnmt1, Dnmt2, and Rad8 subfamilies. We find evidence that the domain fusion event between characteristic domains of DNMtase and Snf2 families in the Rad8 family happened exclusively in fungi, indicating the functional novelty of the Rad8 protein in fungi. Furthermore, expression profiles of the predicted DNMtase genes in the mushroom *P. ostreatus* demonstrated that DNMtases have dynamic expression patterns during mushroom growth, indicating that DNMtases are organ and stage specific. Specifically, DNA methyltransferases displayed higher expression levels in dikaryons than in monokaryons. Consistent with the expression profiles, the DNA methyltransferase inhibitor 5-azacytidine was observed to exert more inhibitory effects on the growth of dikaryons than on that of monokaryons. In summary, DNA methyltransferase might play a large role in the growth of mushrooms. In addition, DNA methylation is a dynamic process regulated by both DNA methyltransferases and demethylases (Furner and Matzke, 2011). The identification and characterization of DNA demethylases, and their roles involved in mushroom development are of interest and deserve further investigations.

### DNMtases in *P. ostreatus*

In our study, expression profiles revealed that DNMtases are functionally divergent and may play a crucial role in mushroom development. For example, Dnmt1a and Dnmt1b are expressed at particularly high levels in the veil of before turning to pale compared with the other stages, indicating that these two genes might be important regulators of veil development. By contrast, another Dnmt1 homolog, Dnmt1c, reaches its peak at cap maturation (Figure 4B). These data suggest that although highly conserved in protein sequence, Dnmt1/Masc2 homologs are functionally divergent. Additionally, Rad8 is highly expressed during early fruit body formation (Figure 4B), suggesting that Rad8 might play a part in fruiting body growth and development. Given that meiosis occurs prior to the maturation of tissue in the fruit body (Kües, 2000), it might be that Rad8 is required for MIP which occurs premeiotically.

The expression levels of DNMtases in dikaryons are significantly higher than those in monokaryons (Figure 4B). Our experiment revealed that 5-azacytidine could inhibit the growth of dikaryons. Upon microscopic examination, we found that 5-azacytidine can affect the cell length of dikaryons, resulting in less but longer cell. However, 5-azacytidine does not have similar effects on monokaryons (Figure 5, Supplementary Figure 3). Quantitative PCR data indicate that 5-azacytidine treatment does not affect the transcript levels of any DNMtase genes in either dikaryons or monokaryons (Figure 5C). Considered that 5-azacytidine specifically inhibits the DNMtase enzyme activity post-transcriptionally, it is likely that the reduced DNMtase enzyme activities by 5-azacytidine treatment might directly contribute to the growth defects in dikaryons.

Previous studies have demonstrated that 5-azacytidine can elicit reversible cell cycle arrest in *S. pombe* (ascomycetes) at the G2/M transition by causing DNA damage and inhibiting a late stage of DNA replication (Taylor et al., 1996). In line with the conclusion reached by Taylor et al. (1996) regarding *S. pombe*, 5-azacytidine may also influence cell mitosis in *P. ostreatus* (basidiomycetes), which is consistent with our finding that the treated mycelial colony was smaller than the control (Figure 5A). However, it is noteworthy that the inhibitory effect of 5-azacytidine in *P. ostreatus* is limited to dikaryons, which has not been reported previously in basidiomycetes. The most important difference between monokaryons and dikaryons is that the latter typically contains two genetically distinct haploid nuclei in its cells (Kües, 2000). Considering this difference, we propose that the growth of dikaryons with two nuclei requires more complicated epigenetic regulatory systems such as DNA methylation compared with monokaryons containing only one nucleus. These two primary types of mycelia are distinguished by their life cycles: dikaryons are fertile and can form mushrooms, whereas monokaryons are infertile (Kües, 2000). This also indicates that DNA methylation might play roles in dikaryons differentiation and mushroom formation, consistent with our dynamic expression profiles during the growth of *P. ostreatus* (Figure 4).

The observation that 5-azacytidine treatment can greatly elicit secondary metabolites in dikaryons but not in monokaryons could be explained by direct or indirect effects of the DNA methylation inhibition. It is reported that 5-azacytidine can directly activate silent gene clusters required for the biosynthesis of secondary metabolites which are repressed by DNA methylation under standard laboratory conditions (Brakhage and Schroeckh, 2011). On the other hand, secondary metabolites could accumulate under stress conditions (Ochiai et al., 2007). Considered that monokaryons grow well while dikaryons exhibit growth defect under 5-azacytidine treatment, it might be the case that malfunction of DNA methylation could act as a stress to indirectly activate the accumulation of secondary metabolites. Taken together, the expression profiles and 5-azacytidine treatment experiment would provide a foundation for understanding how DNA methylation is involved in the growth of dikaryons and mushrooms.

### Reorganization of domains and evolutionary novelty

In *A. immerses*, the *de novo* methylation process MIP is initiated by Masc1 and reversibly methylates C residues in repetitious sequences during sexual reproduction (Fuks et al., 2000). *De novo* methylation of duplicated sequences in *C. cinereus* is similar to MIP, although the general level of methylation is low and gene inactivation is rare compared to *A. immerses* (Freedman and Pukkila, 1993). However, no Masc1 homologs have been identified in *C. cinereus* to date (Zemach and Zilberman, 2010). The newly identified Rad8 DNMTase proteins may be required for *de novo* methylation in *C. cinereus*.

Snf2 family proteins are responsible for chromatin remodeling by consuming energy provided by ATP (Flaus, 2006). In particular, a plant-specific Swi2/Snf2 homolog named DRD1 is required for RdDM (RNA directed DNA methylation) (Huettel et al., 2007). The fact that no DRD1 orthologs have been identified outside the plant kingdom indicates that other Snf2 proteins might play a similar role in other species (Kanno et al., 2004). The fungi-specific domain fusion event between the characteristic domains of DNMtase and Snf2 families might lead to functional combination (Figure 3). It may be the case that Rad8 proteins not only act as DNMtases but also play the same role as DRD1 in fungi. Future biochemical and genetic investigations may provide more evidence to strengthen this hypothesis.

## Conclusion

Mushrooms are highly valued for agricultural uses of their fruiting bodies. The mechanism by which DNA methylation involves in mushroom development has not been investigated previously. In our study, genes encoding DNA methyltransferase were identified in representative fungal species, especially in basidiomycetes. Expression analysis and drug treatment experiments suggest that DNMtases may be involved in the mushroom growth and development of mushroom. We believe that the work presented here would broaden our understanding about the regulatory and biological significance of DNA methylation in basidiomycetes.

## Author contributions

RH, TG, and YL wrote the main text and prepared the figures and tables. TG and YL designed the project. RH, QD, YX performed the experiments and the analysis. All authors reviewed and edited the manuscript.

## Funding

This work was supported by Fundamental Research Funds for the Central Universities (KYZ201605) to TG.

### Conflict of interest statement

The authors declare that the research was conducted in the absence of any commercial or financial relationships that could be construed as a potential conflict of interest.
