# Corrigendum: Comparative Analysis of DNA Methyltransferase Gene Family in Fungi: A Focus on Basidiomycota

**DOI:** 10.3389/fpls.2017.00123

**Published:** 2017-02-03

**Authors:** Ruirui Huang, Qiangqiang Ding, Yanan Xiang, Tingting Gu, Yi Li

**Affiliations:** ^1^State Key Laboratory of Plant Genetics and Germplas Enhancement and College of Horticulture, Nanjing Agricultural UniversityNanjing, China; ^2^Laboratory of Plant Hormone, College of Life Sciences, Nanjing Agricultural UniversityNanjing, China; ^3^Department of Plant Science and Landscape Architecture, University of ConnecticutStorrs, CT, USA

**Keywords:** DNA methyltransferase, basidiomycetes, *Pleurotus ostreatus*, mushroom development, DNA methyltransferase inhibitor

Reason for Corrigendum:

There was an error in the literature cited for the use of ACTIN as the reference gene for the qPCR analysis in “Materials and Methods” section. In the sentence “Results were analyzed by using the ΔΔCT (Livak and Schmittgen, 2001) method using ACTIN (Castanera et al., 2012) as the internal control.”, “Castanera et al., 2012” should be replaced with “Fernández-Fueyo et al., 2014; Pezzella et al., 2013.” However, the original error or the corrigendum does not change the scientific conclusions of the publication.

In addition, in the original article we have neglected to thank the financial support from National Natural Science Foundation of China, grant number 31672123 to TG. The authors apologize for this oversight.

## Conflict of interest statement

The authors declare that the research was conducted in the absence of any commercial or financial relationships that could be construed as a potential conflict of interest.
