# Supplementary material for: Comparative Analysis of DNA Methyltransferase Gene Family in Fungi: A Focus on Basidiomycota
Source: Front Plant Sci. 2016 Oct 21;7:1556. doi: 10.3389/fpls.2016.01556 (PMC5073141; doi:10.3389/fpls.2016.01556)
Supplement: Supplementary file 1 [file Table1.PDF]

Species	DNMT1			DNMT2			Rad8			DNMT3		
	Gene_ID	Protein number	Name	Gene_ID	Protein number	Name	Gene_ID	Protein number	Name			
Agaricus bisporus	18076563	XP_006458036.1	AbDnmt1a	18083664	XP_006454830.	AbDnmt2	18079820	XP_006461865.	AbRad8			
	18076367	XP_006453977.1	AbDnmt1b									
Coprinopsis cinerea	6009669	XP_001833175.2	CcDnmt1a	6004942	XP_001828513.	CcZMET4	6007796	XP_001831325.	CcRad8			
	3286518	XP_001829400.2	CcDnmt1b									
Galerina marginata	4287	KDR81704.1	GmDnmt1a	5152	KDR80763.1	GmDnmt2	5085	KDR80696.1	GmRad8			
	826	KDR85517.1	GmDnmt1b									
Laccaria bicolor	6070896	XP_001875435.1	LbDnmt1a	6069082	XP_001873676.	LbDnmt2	6070636	XP_001874556.	LbRad8			
	6069549	XP_001873517.1	LbDnmt1b									
Moniliophthora roreri	19296591	XP_007846566.1	MrDnmt1a	19283455	XP_007852198.	MrDnmt2	19291201	XP_007855526.	MrRad8			
	19289230	XP_007843483.1	MrDnmt1b									
	19295290	XP_007844490.1	MrDnmt1c									
Pleurotus ostreatus	580	KDQ33030.1	PoDnmt1a	3100	KDQ30360.1	PoDnmt2	7450	KDQ26584.1	PoRad8			
	3147	KDQ30406.1	PoDnmt1b									
	4110	KDQ31353.1	PoDnmt1c									
Schizophyllum commune	9587380	XP_003034892.1	ScDnmt1a	9593277	XP_003026560.	ScDnmt2	9590980	XP_003033430.	ScRad8			
	9589029	XP_003037017.1	ScDnmt1b									
Ceriporiopsis subvermispora	3941	EMD38163.1	CsDnmt1a				3566	EMD39297.1	CsRad8			
	9848	EMD32847.1	CsDnmt1b									
Dichomitus squalens	18845352	XP_007361443.1	DsqDnmt1a	18843926	XP_007371548.	DsqDnmt2	18841321	XP_007370022.	DsqRad8			
	18840601	XP_007362279.1	DsqDnmt1b									
Trametes versicolor	19410705	XP_008036653.1	TvDnmt1	19410338	XP_008033359.	TvDnmt2	19407974	XP_008038755.	TvRad8			
Wolfiporia cocos*	2012	135973	WcDnmt1				10306	166855	WcRad8			
Postia placenta	8145442	XP_002472324.1	PpDnmt1a				8144819	XP_002471603.	PpRad8			
	8142130	XP_002474649.1	PpDnmt1b									
Fomitopsis pinicola	7762	EPS98538.1	FpDnmt1	8244	EPS98063.1	FpDnmt2	3305	EPT02999.1	FpRad8			
Phanerochaete carnosar	18910184	XP_007395598.1	PcDnmt1a	18909401	XP_007389852.	PcDnmt2	18913385	XP_007400909.	PcRad8			
	18910185	XP_007395604.1	PcDnmt1b									
	18912289	XP_007390923.1	PcDnmt1c									
Coniophora puteana	19204699	XP_007764560.1	CpDnmt1b	19202492	XP_007763121.	CpDnmt2a	19198662	XP_007771057.	CpRad8			
	19209733	XP_007775686.1	CpDnmt1c									
	19211763	XP_007763763.1	CpDnmt1a									
Serpula lacrymans	18813589	XP_007322072.1	SlDnmt1a	18816933	XP_007323674.	SlDnmt2						
	18814073	XP_007313106.1	SlDnmt1b									
Jaapia argillacea	4595	KDQ61172.1	JaDnmt1a	10605	KDQ54985.1	JaDnmt2	3458	KDQ61535.1	JaRad8			
	1429	KDQ62886.1	JaDnmt1b									
Auricularia delicata	18853812	XP_007338776.1	AdDnmt1a	18852618	XP_007336601.	AdDnmt2	18845888	XP_007350196.	AdRad8			
	18853274	XP_007338925.1	AdDnmt1b									
	18849741	XP_007353728.1	AdDnmt1c									
Botryobasidium botryosum	2643	KDQ19575.1	BbDnmt1	4435	KDQ17706.1	BbDnmt2	4425	KDQ17696.1	BbRad8			
	8760	KDQ13404.1	BbMasc1like									
	5732	KDQ16327.1	BbDim-2									
Fomitiporia mediterranea	18674831	XP_007267671.1	FmDnmt1a	18680128	XP_007266076.	FmDnmt2	18669863	XP_007262527.	FmRad8			
	18676869	XP_007267672.1	FmDnmt1b									
	18677243	XP_007271194.1	FmDnmt1c									
Piriformospora indica	9709	CCA68417.1	PiDnmt1a	11387	CCA66740.1	PiDnmt2	5525	CCA72586.1	PiRad8			
	5793	CCA72316.1	PiDnmt1b									
Heterobasidion annosum*	12382	432600	HaDnmt1a	13395	306746	HaDnmt2	2038	124547	HaRad8			
	2987	442857	HaDnmt1b									
Stereum hirsutum	18925465	XP_007301310.1	ShDnmt1a	18925465	XP_007298544.	ShDnmt2	18807105	XP_007304058.	ShRad8			
	18805144	XP_007300459.1	ShDnmt1b									
	18795663	XP_007308633.1	ShMasc1like									
Dacryopinax sp.	2067	EJU05027.1	DsDnmt1				10132	EJT96648.1	DsRad8			
Gloeophyllum trabeum	19303607	XP_007863211.1	GtDnmt1a	19305369	XP_007860231.	GtDnmt2	19300063	XP_007865021.	GtRad8			
	19307784	XP_007860485.1	GtDnmt1b									
Puccinia graminis	10540608	XP_003322205.2	PgDnmt1				10530779	XP_003335291.	PgRad8			
Malassezia globosa												
Wallemia sebi*												
Melampsora larici	18934023	XP_007409499.1	MlDnmt1				18933206	XP_007415362.	MlRad8			
Ustilago maydis												
Saccharomyces cerevisiae												
Schizosaccharomyces pombe				2540766	NP_595687.1	SpPmt1						
Neurospora crassa	3876054	XP_959891.1	NcDim-2									
	23568375	XP_011392925.1	NcRID									
Ascobolus immersus*	4118	412515	AiMasc2				16663	333534	AiRad8			
	4553	37441	AiDim-2									
	15137	243387	AiMasc1									
Aspergillus clavatus	4702168	XP_001270179.1	AcDmtA				4700154	XP_001267835.	AcRad8			
Aspergillus flavus	7920717	XP_002378378.1	AfDmtA									
Blastomyces dermatitidis	8509447	XP_002624554.1	BdDmtA									
Phycomyces blakesleeanus*	3098	77038	PbDnmt1	8823	146309	PbDnmt2						
	5627	132318	PbDim-2									
Homo sapiens	1786	NP_001124295.1	HsDnmt1	1787	NP_004403.1	HsDnmt2				1789	NP_008823.1	HsDnmt3B
										1788	NP_072046.2	HsDnmt3A

Supplementary Table 1 A list of accession numbers for DNA methylase domain-containing proteins used for analyses. "*" represents this sequence was downloaded from the JGI; the others were downloaded from the NCBI.
