# Supplementary material for: Comparative Analysis of DNA Methyltransferase Gene Family in Fungi: A Focus on Basidiomycota
Source: Front Plant Sci. 2016 Oct 21;7:1556. doi: 10.3389/fpls.2016.01556 (PMC5073141; doi:10.3389/fpls.2016.01556)
Supplement: Supplementary file 2 [file Table2.DOC]

| **Species** | **Website** | **Version** |
| --- | --- | --- |
| *A.bisporus* | https://www.ncbi.nlm.nih.gov/assembly/GCF_000300575.1 | Agabi_varbisH97_2 |
| *C.cinerea* | https://www.ncbi.nlm.nih.gov/assembly/GCF_000182895.1/ | CC3 |
| *G.marginata* | https://www.ncbi.nlm.nih.gov/assembly/GCA_000697645.1/ | Galma1 |
| *L.bicolor* | https://www.ncbi.nlm.nih.gov/assembly/GCF_000143565.1/ | V1.0 |
| *M.roreri* | https://www.ncbi.nlm.nih.gov/assembly/GCF_000488995.1 | M_roreri_MCA_2997_v1 |
| *P.ostreatus* | https://www.ncbi.nlm.nih.gov/assembly/GCA_000697685.1/ | PleosPC15_2 |
| *S. commune* | https://www.ncbi.nlm.nih.gov/assembly/GCF_000143185.1 | v1.0 |
| *C.subvermispora* | https://www.ncbi.nlm.nih.gov/assembly/GCA_000320605.2/ | Ceriporiopsis subvermispora B v1.0 |
| *D. squalens* | https://www.ncbi.nlm.nih.gov/assembly/GCF_000275845.1/ | Dichomitus squalens v1.0 |
| *T. versicolor* | https://www.ncbi.nlm.nih.gov/assembly/GCF_000271585.1/ | Trametes versicolor v1.0 |
| *W.cocos** | http://genome.jgi.doe.gov/Wolco1/Wolco1.download.html | Wolfiporia cocos MD-104 SS10 |
| *P. placenta* | https://www.ncbi.nlm.nih.gov/assembly/GCF_000006255.1/ | Postia placenta V1.0 |
| *F. pinicola* | https://www.ncbi.nlm.nih.gov/assembly/GCA_000344655.2/ | Fompi3 |
| *P. carnosar* | https://www.ncbi.nlm.nih.gov/assembly/GCF_000300595.1 | Phanerochaete carnosa HHB-10118-Sp v1.0 |
| *C. puteana* | https://www.ncbi.nlm.nih.gov/assembly/GCF_000271625.1/ | Conpu1 |
| *S. lacrymans* | https://www.ncbi.nlm.nih.gov/assembly/GCF_000218685.1 | v1.0 |
| *J. argillacea* | https://www.ncbi.nlm.nih.gov/assembly/GCA_000697665.1/ | Jaaar1 |
| *A. delicata* | https://www.ncbi.nlm.nih.gov/assembly/GCF_000265015.1/ | SS-5 V1.0 |
| *B. botryosum* | https://www.ncbi.nlm.nih.gov/assembly/GCA_000697705.1/ | Botbo1 |
| *F. mediterranea* | https://www.ncbi.nlm.nih.gov/assembly/GCF_000271605.1/ | Fomme1 |
| *P. indica* | https://www.ncbi.nlm.nih.gov/assembly/GCA_000313545.1/ | ASM31354v1 |
| *H. annosum** | http://genome.jgi.doe.gov/Hetan2/Hetan2.download.html | Heterobasidion irregulare TC 32-1 |
| *S. hirsutum* | https://www.ncbi.nlm.nih.gov/assembly/GCF_000264905.1/ | Stehi1 |
| *D. sp.* | https://www.ncbi.nlm.nih.gov/assembly/GCA_000292625.1/ | Dacryopinax sp. DJM 731 SSP1 v1.0 |
| *G. trabeum* | https://www.ncbi.nlm.nih.gov/assembly/GCF_000344685.1/ | Glotr1_1 |
| *P. graminis* | https://www.ncbi.nlm.nih.gov/assembly/GCF_000149925.1/ | ASM14992v1 |
| *M. globosa* | https://www.ncbi.nlm.nih.gov/assembly/GCF_000181695.1 | ASM18169v1 |
| *W. sebi** | http://genome.jgi.doe.gov/Walse1/Walse1.download.html | v1.0 |
| *M. larici* | https://www.ncbi.nlm.nih.gov/assembly/?term=Melampsora+larici | v1.0 |
| *U. maydis* | https://www.ncbi.nlm.nih.gov/assembly/GCF_000328475.2 | Umaydis521_2.0 |
| *S. cerevisiae* | https://www.ncbi.nlm.nih.gov/assembly/GCF_000146045.2 | R64 |
| *S. pombe* | https://www.ncbi.nlm.nih.gov/assembly/GCF_000002945.1/ | ASM294v2 |
| *N. crassa* | https://www.ncbi.nlm.nih.gov/assembly/GCF_000182925.2 | NC12 |
| *A. immersus** | http://genome.jgi.doe.gov/pages/dynamicOrganismDownload.jsf?organism=Ascim1 | v1.0 |
| *A. clavatus* | https://www.ncbi.nlm.nih.gov/assembly/GCF_000002715.2/ | ASM271v1 |
| *A. flavus* | https://www.ncbi.nlm.nih.gov/assembly/?term=Aspergillus+flavus | JCVI-afl1-v2.0 |
| *B. dermatitidis* | https://www.ncbi.nlm.nih.gov/assembly/GCF_000003855.2/ | ASM385v2 |
| *P.blakesleeanus** | http://genome.jgi.doe.gov/pages/dynamicOrganismDownload.jsf?organism=Phybl2 | v2.0 |
| *H. sapiens* | https://www.ncbi.nlm.nih.gov/assembly/GCF_000306695.2 | CHM1_1.1 |

**Supplementary Table 2** The genome assembly version and corresponding web link of relevant genome.
