# Supplementary material for: Comparative Analysis of DNA Methyltransferase Gene Family in Fungi: A Focus on Basidiomycota
Source: Front Plant Sci. 2016 Oct 21;7:1556. doi: 10.3389/fpls.2016.01556 (PMC5073141; doi:10.3389/fpls.2016.01556)
Supplement: Supplementary file 3 [file Table3.PDF]

Kingdom	Phylum of clade	Species	DNA_methyltransferase	Helicase_C	SNF2_N	Helicase_C+SNF2_N
Protista	Algae-like	C.merolae	1	57	15	11
		C.reinhardtii	7	95	28	13
		T.thermophila	0	85	25	25
Eubacteria		E.coli	1	20	3	0
		S.coelicolor	1	28	6	2
		R.opacus	0	20	8	8
		T.erythraeum	0	27	7	7
Plantae	Bryophyta	P.patens	5	147	62	48
	Angyospermophyta	A.thaliana	10	209	77	45
		P.trichocarpa	5	222	92	68
	Gymnospermophyta	P.abies	6	160	63	45
Animalia	Protomia	N.vectensis	2	108	32	23
		C.elegans	0	121	40	28
	Echinodermata	S.purpuratus	3	182	55	44
	Chordata	Homo sapiens	4	237	89	53
Archaebacteria		N.pharaonis	0	13	3	0
		M.acetivorans	0	17	4	1
		S.tokodaii	1	14	5	0
Fungi	Basidiomycota	C.cinerea	4	26	67	23
		P.ostreatus	5	29	91	22
	Ascomycota	A.flavus	2	20	52	26
		S.cerevisiae	0	17	77	17
	Mucoromycotina	P.blakesleeanus	3	19	53	16

Table S3. Genes encoding DNA methylase/SNF2_N/Helicase_C domains identified in a genome-wide survey.
