# Supplementary material for: Comparative Analysis of DNA Methyltransferase Gene Family in Fungi: A Focus on Basidiomycota
Source: Front Plant Sci. 2016 Oct 21;7:1556. doi: 10.3389/fpls.2016.01556 (PMC5073141; doi:10.3389/fpls.2016.01556)
Supplement: Supplementary file 4 [file Table4.PDF]

Gene Name	Oligonucleotides Sequences
Actin-Forward primer	AGTCGGTGCCTTGGTTAT
Actin-Reverse primer	ATACCGACCATCACACCT
PoDnmt1a-Forward primer	GTACCCTTACCTTCCGATGAAC
PoDnmt1a-Reverse primer	CTCGTGAATGTGGCCGTATAA
PoDnmt1b-Forward primer	TGGCCACTATCAAGAGCATTAG
PoDnmt1b-Reverse primer	AGTATCCCTTGACTCTCCCTAG
PoDnmt1c-Forward primer	GGATTTGTTGTGGTTGGGTATG
PoDnmt1c-Reverse primer	CAACTTTACGAACGGCTGATTT
PoRad8-Forward primer	CTTGACGCTTTAGGATGGAGAG
PoRad8-Reverse primer	GTCGATGAGGGCTAGTGTAATG

Table S4. All of the primers used in this experiment.
