# Supplementary material for: Comparative Analysis of DNA Methyltransferase Gene Family in Fungi: A Focus on Basidiomycota
Source: Front Plant Sci. 2016 Oct 21;7:1556. doi: 10.3389/fpls.2016.01556 (PMC5073141; doi:10.3389/fpls.2016.01556)
Supplement: Supplementary file 5 [file Image1.PDF]

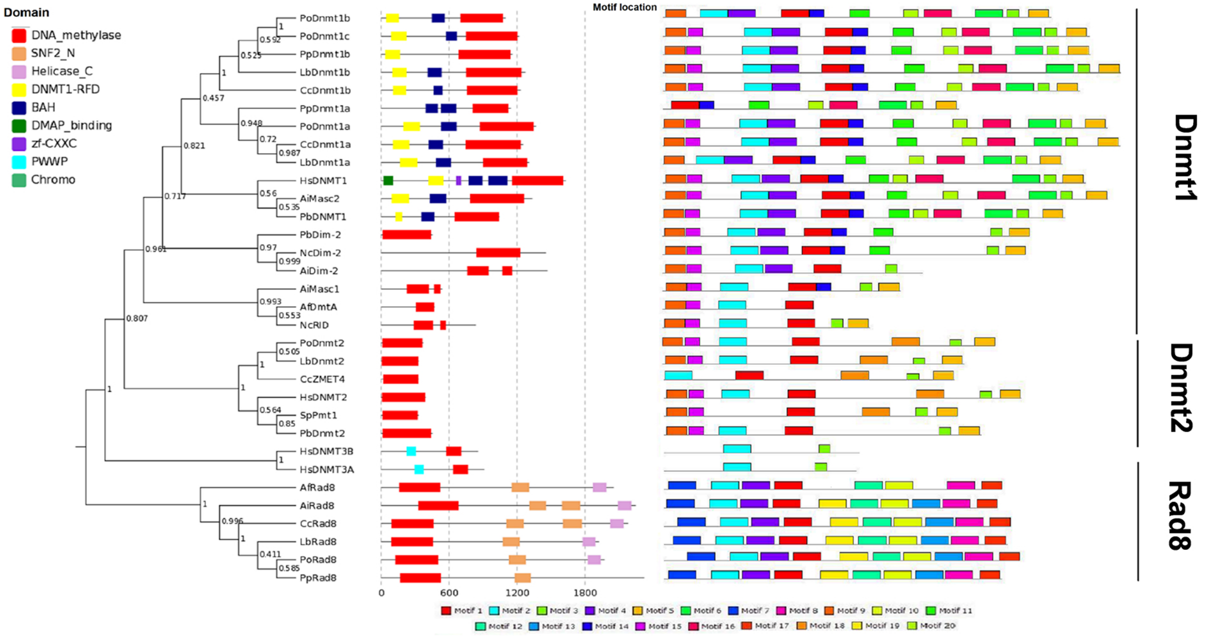

Supplementary Figure 1 Phylogenetic relationship, domain architecture and motif distribution of Mtases identified in 10 representative species investigated. Maximum likelihood tree of identified 32 different proteins was constructed based on the conserved catalytic domains using MEGA6.0. The bootstrap values of each branch points are indicated. The domain architecture analysis of the identified proteins was performed by the Pfam database. The schematic representation of the conserved motifs in the DNMTases detected by MEME analysis. Each motif is represented by a color box numbered at the Bottom.
