# Supplementary figures and images for: Comparative Analysis of DNA Methyltransferase Gene Family in Fungi: A Focus on Basidiomycota
Source: Front Plant Sci. 2016 Oct 21;7:1556. doi: 10.3389/fpls.2016.01556 (PMC5073141; doi:10.3389/fpls.2016.01556)

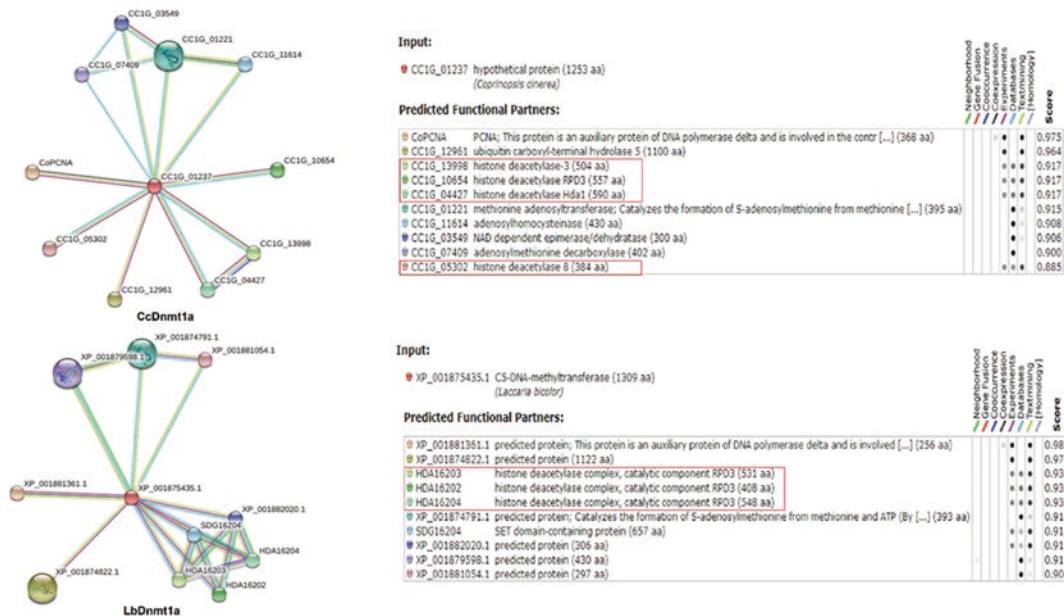

**Fig. S2.** Protein interaction works of Dnmt1/Msc2 identified in *C. cinerea* and *L. bicolor*.

Supplement: Supplementary file 6 [file Image2.PDF]

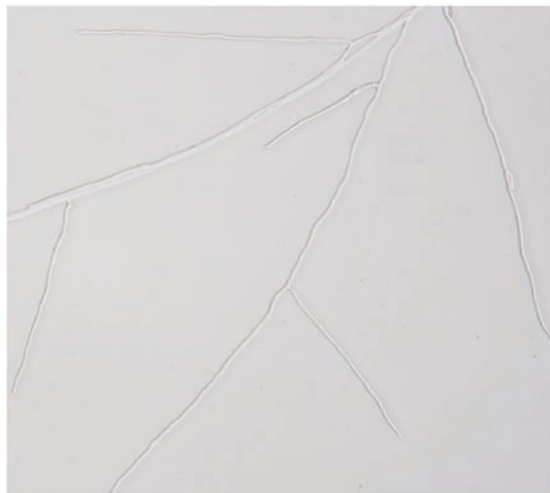

**MOCK**

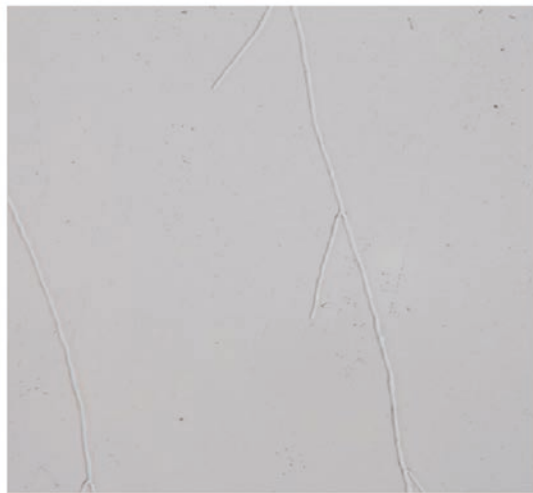

**250uM**

**Fig. S3** Micrographs of terminal monocaryon treated with 250uM 5-azacytidine and the control.

Supplement: Supplementary file 7 [file Image3.PDF]
